# Pacemaker-mediated tachycardia as a pacing maneuver for arrhythmia diagnostics

**DOI:** 10.1016/j.hrcr.2025.11.023

**Published:** 2025-12-04

**Authors:** Thomas Seiler, Andreas Haeberlin, Fabian Noti, Boldizsar Kovacs

**Affiliations:** Department of Cardiology, Inselspital, Bern University Hospital, University of Bern, Bern, Switzerland

**Keywords:** CRT, PMT, Loss of LV pacing, Arrhythmia, LV sensing algorithm

**Key Teaching Points**

• Pacemaker-mediated tachycardia induced during ongoing tachyarrhythmias can be useful as diagnostic pacing maneuver in the differential diagnosis of arrhythmias.
• In Biotronik CRT devices, left ventricular (LV) sensing with an activated T-wave protection algorithm helps to detect and understand desynchronization episodes, but it may also cause unintended loss of LV pacing.
• Unintended loss of LV-pacing can be addressed by shortening the left ventricular upper rate interval to a minimum of 375ms or by disabling LV-sensing.

## Introduction

A 78-year-old man with ischemic cardiomyopathy and a severely reduced left ventricular ejection fraction of 20% underwent cardiac resynchronization therapy (CRT) with a defibrillator implantation 3 years ago with >98% biventricular (BiV) pacing in all his follow-ups. Relevant comorbidities include chronic kidney disease (Kidney Disease: Improving Global Outcomes) grade 4 and arterial hypertension. For heart failure management, he received treatment with an angiotensin receptor–neprilysin inhibitor, a beta-blocker, and a sodium-glucose cotransporter 2 inhibitor. Owing to chronic fatigue and low blood pressure, his primary care physician reduced the beta-blocker (nebivolol, from 10 mg to 5 mg). Several weeks later, remote monitoring revealed a decline in BiV pacing to 76%, prompting a visit to our outpatient clinic. In this article, we describe a novel mechanism that aids in differentiating the differential diagnosis of device-detected arrhythmias.

Initial testing of his CRT with a defibrillator (Biotronik Acticor 7 HF-T QP) showed a normal device function with stable lead parameters and a paced QRS width of 95 ms.

**Figure 1:**
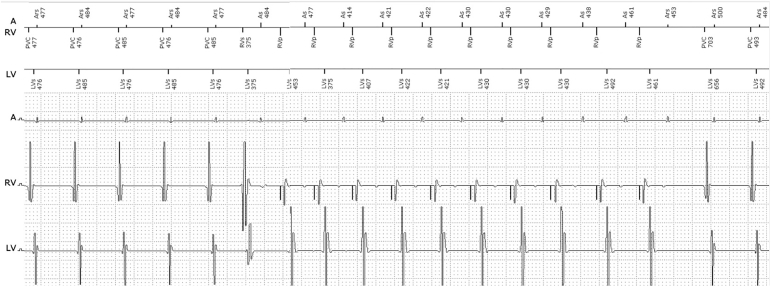
Unknown tracing.

Pertinent device settings are presented in Table 1. The device arrhythmia log showed several CRT-interrupt sequences related to ventriculoatrial (VA)-sequential tachyarrhythmias with a cycle length of ∼480 ms (Figure 1).

**Table 1:** Device programming

Device settings			
Bradycardia			
Mode	DDD		
Base rate [/min]	50		
Upper rate limit [/min]	130		
AV delay (paced/sensed) [ms]	100/80		
PVARP	225		
PVARP after PVC	350		
PVAB	80		
Auto-adapt CRT	OFF		
T-wave protection	On		
LVURI	400 ms		
Tachycardia			
VT1	154/min	Monitor	
VF	200/min	ATP during charging, 40 J, 40 J, 4 × 40 J	

ATP = antitachycardia pacing; AV = atrioventricular; CRT = cardiac resynchronization therapy; LVURI = left ventricular upper tracking interval; PVAB = postventricular atrial blanking; PVARP = postventricular atrial refractory period; PVC = premature ventricular contraction; VF = ventricular fibrillation; VT1 = ventricular tachycardia type 1.

## Discussion

**Figure 2:**
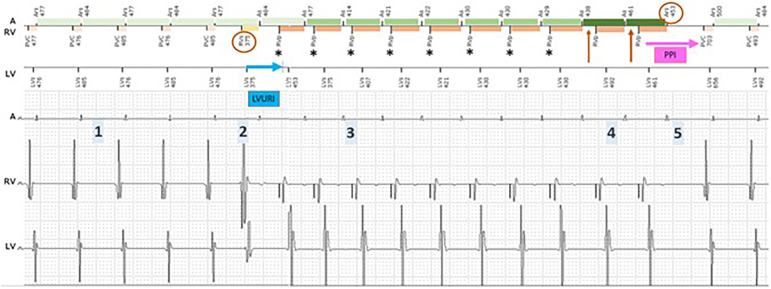
Displayed is a VA-sequential tachycardia and a diagnostic ventricular entrainment maneuver provoked by a pacemaker-mediated tachycardia (PMT). (1) The start and end of the episode were not recorded by the device. A VA-sequential tachycardia with a cycle length of ∼477 ms (125 bpm), where V-V leads A-A, with a relatively short VA interval of ∼80 ms (within the PVARP) is registered as a CRT-interrupt sequence. The RV signal is declared as a “PVC,” despite having the same morphology compared with the intrinsic rhythm. Given that the atrial signal falls into the PVARP, it is not tracked, which leads to desynchronization. (2) An early, possibly His-refractory PVC occurs (*first red circle*), without changing the subsequent A-A-delay, ruling out VA conduction of this beat. This PVC is marked as a normal beat by the device. The early PVC in turn leads to the “unmasking” of the next atrial signal by “pulling in” the PVARP allowing an AS event and tracking at the RV upper rate limit. (3) With RV pacing, the (retrograde) VA conduction prolongs to ∼320 ms and falls again outside the PVARP, which provokes a PMT again with loss of BiV pacing. The reason for desynchronization is an activated LV sensing and the Biotronik T-wave protection algorithm designed to prevent pacing during the vulnerable period of the left ventricle. Any Vs, Vp, or inhibited Vp on the LV channel initiates an LV upper tracking interval (LVURI), during which no LV pacing stimulus can be delivered. Given that the RVp (*asterisks*) is delivered before the ending of the LVURI (*blue arrow*), LVp is withheld and intrinsic RV to LV conduction is sensed on the LV channel ∼80 ms later. (4) After 8 consecutive beats with stable VP-AS intervals (*asterisk*) with a length shorter than the VA criterion (which is nominally set at 350 ms), the device suspects a PMT. To confirm the diagnosis, the AV delay is automatically prolonged by 50 ms for the next 2 beats (*red arrow*). If the VA delay remains stable, the PMT is confirmed and the PVARP is extended according to the VA interval + 50 ms. (5) At the next beat, PVARP extension results in termination of the PMT (*red circle*). The response after the last ventricular paced beat is a VAV response with a long PPI-TCL of 780 ms, being 240 ms longer than the PMT cycle length, favoring the diagnosis of AVNRT or junctional tachycardia. A-A = atrial-to-atrial; AVNRT = atrioventricular nodal reentrant tachycardia; BiV = biventricular; bpm = beats per minute; LV = left ventricular; LVp = left ventricular pacing; PPI-TCL = postpacing interval minus tachycardia cycle length; PVARP = postventricular atrial refractory period; PVC = premature ventricular contraction; RV = right ventricular; RVp = right ventricular pacing; VA = ventriculoatrial; VAV = ventricular-atrial-ventricular; V-V = ventricular-to-ventricular.

Low BiV pacing is a frequent clinical problem among CRT patients. To enhance resynchronization, it is crucial to understand the underlying mechanism, where LV sensing can be helpful. In our patient, several asymptomatic episodes of a VA-sequential tachycardia of unknown duration were observed. Considering the VA interval of 80 ms and an unchanged ventricular morphology, the main differential diagnosis includes an atrioventricular nodal reentrant tachycardia (AVNRT), junctional tachycardia, or atrial tachycardia with a long PR interval. Although the relatively short VA delay makes orthodromic AVNRT unlikely, it cannot be completely ruled out. A detailed description and interpretation of the device tracing is shown in Figure 2.

In summary, an unusual and in this case essentially diagnostic finding for further differentiation was an intermittent premature ventricular contraction during supraventricular tachycardia–triggered pacemaker-mediated tachycardia (PMT) with successful termination by the PMT algorithm of the device. This resulted in a ventricular overdrive-pacing maneuver commonly performed during electrophysiological studies. The resulting ventricular-atrial-ventricular response with a long postpacing interval minus tachycardia cycle length supports AVNRT or junctional tachycardia as the primary mechanism for the arrhythmia.^1^

An electrophysiological study and ablation were recommended, but declined by the patient. Therefore, the further management included a change from nebivolol to metoprolol with subsequent uptitration. This led to a significant increase in the BiV pacing to 95%. Although LV sensing and active T-wave protection can provide valuable insights into the mechanisms of desynchronization and support the diagnosis of tachyarrhythmias below the monitor zone, in our patient, they are also responsible for desynchronization during PMT.^2^ To address this issue, the left ventricular upper tracking interval can be shortened from the nominal 400 ms to a minimum of 375 ms. If this adjustment is not sufficient, deactivation of the T-wave protection algorithm and LV sensing may be considered. However, this will prevent the device from detecting recurrent slow AVNRTs, provided that the monitor zone is not programmed at a very low threshold. Another important consideration is that a very short atrioventricular delay may increase the risk of LV pacing when the T-wave protection algorithm is active. Therefore, the atrioventricular delay should be programmed as short as necessary, but not shorter than required.

## Conclusion

PMTs induced during ongoing tachyarrhythmias can be useful as a diagnostic pacing maneuver in the further differential diagnosis of arrhythmias. The Biotronik T-wave protection algorithm helps to detect and understand desynchronization episodes, but it may also cause unintended loss of LV pacing.

## Disclosures

The authors have no conflicts of interest to disclose.

## References

[1] Michaud G.F., Tada H., Chough S. (2001). Differentiation of atypical atrioventricular node re-entrant tachycardia from orthodromic reciprocating tachycardia using a septal accessory pathway by the response to ventricular pacing. J Am Coll Cardiol.

[2] Haeberlin A., Ploux S., Noel A. (2020). Causes of impaired biventricular pacing in cardiac resynchronization devices with left ventricular sensing. Pacing Clin Electrophysiol.

